# Differentiating Smoking-Related Interstitial Fibrosis (SRIF) from Usual Interstitial Pneumonia (UIP) with Emphysema Using CT Features Based on Pathologically Proven Cases

**DOI:** 10.1371/journal.pone.0162231

**Published:** 2016-09-09

**Authors:** Kum Ju Chae, Gong Yong Jin, Hyun Nyeong Jung, Keun Sang Kwon, Hyemi Choi, Yong Chul Lee, Myoung Ja Chung, Ho Sung Park

**Affiliations:** 1 Department of Radiology, Chonbuk National University Medical School and Hospital, Institute of Medical Science, Research Institute of Clinical Medicine, Jeonju, Jeonbuk, South Korea; 2 Department of Preventive Medicine, Chonbuk National University Medical School and Hospital, Institute of Medical Science, Research Institute of Clinical Medicine, Jeonju, Jeonbuk, South Korea; 3 Department of Statistics and Institute of Applied Statistics, Chonbuk National University, Jeonju, Jeonbuk, South Korea; 4 Department of Internal Medicine, Chonbuk National University Medical School and Hospital, Institute of Medical Science, Research Institute of Clinical Medicine, Jeonju, Jeonbuk, South Korea; 5 Department of Pathology, Chonbuk National University Medical School, Research Institute of Clinical Medicine, Jeonju, Jeonbuk, South Korea; Washington State University, UNITED STATES

## Abstract

**Objective:**

To differentiate smoking-related interstitial fibrosis (SRIF) from usual interstitial pneumonia (UIP) with emphysema on CT in combined pulmonary fibrosis and emphysema (CPFE) patients.

**Materials and Methods:**

This study was approved by the institutional review board and informed consent was waived. We included 65 patients who underwent lung biopsy under the suspicion of UIP pattern on HRCT, and after radiologic-pathologic correlation, they were divided into three groups: UIP without emphysema (n = 30), UIP with emphysema (n = 26), and SRIF (n = 9). The quantitative extent of emphysema in the entire lung was visually assessed and fibrotic patterns were qualitatively analyzed based on six characteristics (asymmetry, juxta-subpleural sparing, emphysema beside the honeycombing area, absence of ground grass attenuation/reticulation in honeycombing area, inhomogeneous honeycombing, and absence of honeycombing in the upper lobes). Kaplan-Meier analysis was used for survival analysis, and logistic regression with a receiver operating characteristic curve was used to predict the possibility of SRIF.

**Results:**

In qualitative analysis of fibrotic patterns, SRIF tended to exhibit more than three of six fibrotic features, whereas UIP with emphysema demonstrated about two of these characteristics (*p* = 0.035). In addition, SRIF had a higher extent of emphysema than UIP with emphysema when they have same amount of fibrosis (*p* = 0.014). In patients with SRIF, 5-year survival rate was 85.7%, while it was 40.7% in UIP with emphysema patients (*p* = 0.035).

**Conclusion:**

Fibrotic CT patterns and survival rate differed between SRIF and UIP with emphysema among CPFE patients, which explains the variable prognosis of CPFE.

## Introduction

Although new information has accumulated on combined pulmonary fibrosis and emphysema (CPFE) regarding the classification of idiopathic interstitial pneumonia (IIP) as a kind of coexisting pattern with a heterogeneous population of patients not believed to represent distinctive IIP, definition of CPFE is still unclear [1]. Radiologically, CPFE is recognized and characterized by the presence of emphysema predominantly in the upper lobes and parenchymal fibrosis in the lower lobes on CT [2, 3], and clinically, CPFE mainly appears in men with a heavy smoking history and appears to be connected with lung cancer due to the composite effect of smoking, emphysema, and pulmonary fibrosis [3, 4].

Although there are reports about pathologic features of smoking-related interstitial fibrosis (SRIF) [5–7], pathologic diagnosis of CPFE is not well-established, and diagnosis of CPFE is focused on radiologic findings. Because radiologic diagnosis of CPFE is wide enough to include both usual interstitial pneumonia (UIP) patients have underlying emphysema and patients with SRIF, the prognosis and survival rates in CPFE could be vary greatly in the literature [8–12]. To important, because SRIF and UIP with emphysema are clinically different disease entities, studies are needed to differentiate SRIF from UIP with emphysema in radiology. Thus we hypothesized that the semi-quantitatively analyzed emphysema extent and fibrosis could be helpful in differentiating SRIF from UIP with emphysema among CPFE patients on CT.

## Materials and Methods

### Patient Selection

This retrospective study was approved by our institutional review board, and informed consent was waived. A total of 116 patients who underwent open lung biopsy or video-assisted thoracoscopic surgery (VATS) biopsy to prove interstitial lung disease (ILD) in our hospital from 2004 through December 2010 were reviewed. A pulmonary pathologist (M.J.C., with 13 years of experience) reviewed the pathologic specimens according to American Thoracic Society-European Respiratory Society guidelines [13]. Among them, 56 patients were diagnosed as UIP which includes pathologically typical (n = 39), possible or probable UIP (n = 17). Nine patients were diagnosed as SRIF which was defined as a mixture of uniform appearing collagen-type alveolar septal fibrosis combined with emphysema with or without some fibroblastic foci, and this did not fit with a named interstitial lung disease [5]. 51 patients were excluded by following reasons, non-specific interstitial pneumonia (NSIP) (n = 16), organizing pneumonia (n = 7), hypersensitivity pneumonitis (HP) (n = 4), respiratory bronchiolitis (RB) (n = 2), bronchiectasis (n = 4), unknown etiology of lung fibrosis (n = 6), connective tissue disease induced ILD (n = 5), and outside CT (n = 7). Thus 65 patients with UIP (n = 56) and SRIF (n = 9) were finally included.

**Fig 1:**
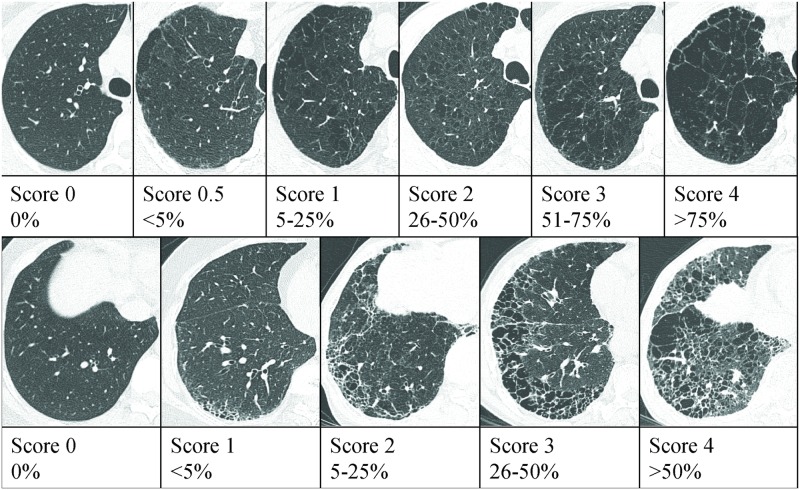
CT images for standard reference cases of emphysema and fibrosis. **(A)** The extent of emphysema was visually scored on a six-point scale. **(B)** The extent of fibrosis was also visually assessed on a five-point scale for reticulation, honeycombing, and total fibrosis.

To evaluate the extent of emphysema in upper and middle lobe and fibrotic interstitial lung abnormality (FILA) in both lower lobes, two radiologists (S.B.C. and Y.S.L., with 12 and 8 years of experience, respectively) reviewed preoperative CT images independently and were blinded to clinical information. We defined honeycomb pattern or reticular opacity with/without ground glass attenuation (GGA) as FILA. The CT findings were interpreted on the basis of the recommendations of the nomenclature committee of the Fleischner Society [14]. Visual assessment was performed by modification of prior methods [15–17]. The extent of emphysema was estimated by using a six-point scale for each lobe as follows: 0 (no emphysema), 0.5 (trivial, <5%), 1 (mild, 5–25%), 2 (moderate, 26–50%), 3 (marked, 51–75%), or 4 (severe, >75%). To avoid the right lung to be valued more heavily than the left, the lingular segment of left lung was evaluated as a separate lobe. We averaged the value obtained from four lobes (both upper lobe, right middle, and left lingular segment). The extent of FILA in both lower lobes was also assessed using a five-point scale for reticulation, honeycombing, or the total extent of those findings in both lower lobes as follows: 0 (no abnormality), 1 (mild, <5%), 2 (moderate, 5–25%), 3 (marked, 26–50%), or 4 (severe, >50%) (Fig 1). A third reader, a chest specialist (G.Y.J., with 15 years of experience) was blinded to the interpretations of previous readers and provided the deciding opinion on scans that were discordantly scored. After radiologic review, 30 patients had a radiologic UIP pattern without emphysema, 35 patients had UIP pattern with emphysema.

**Fig 2:**
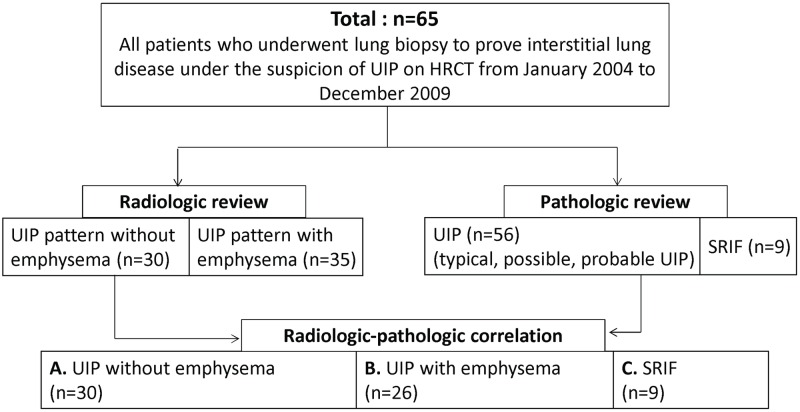
Flowchart of patient selection. UIP = usual interstitial pneumonia, SRIF = smoking-related interstitial fibrosis.

After radiologic-pathologic correlation, 65 patients were divided into three groups: pathologically proven UIP without radiologic emphysema (Group A, n = 30), pathologically proven UIP with radiologic emphysema (Group B, n = 26), and SRIF (Group C, n = 9) (Fig 2).

### Qualitative Imaging Analysis

**Fig 3:**
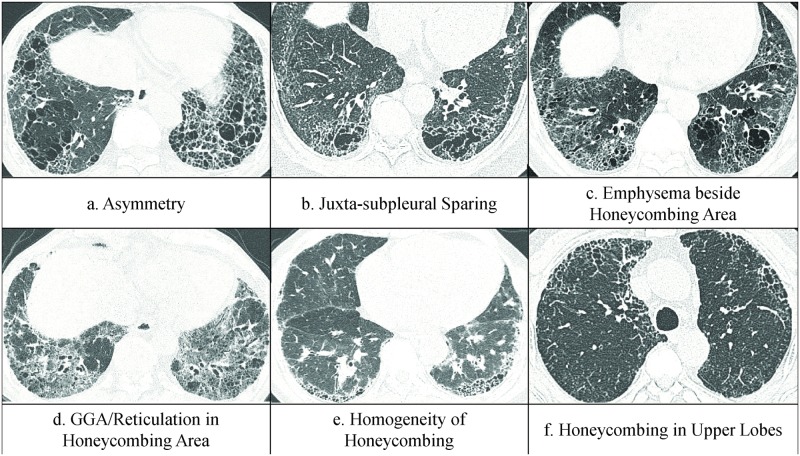
Six standard reference CT images for characterization of FILA. FILA = fibrotic interstitial lung abnormality.

Two radiologists (K.J.C and Y.S.K., with 4 and 3 years of experience, respectively) who were trained in differentiating fibrotic lung disease by CT for 3 years qualitatively analyzed FILA, and a third reader, a chest specialist (G.Y.J. with 15 years of experience) provided a final score for the scans that had been discordantly evaluated. All three readers were blinded to clinical and pathologic information, and the third reader was blinded to the interpretations of previous readers. FILA on both lower lobes in all patients were qualitatively analyzed based on the following CT findings: (a) asymmetry, (b) juxta-subpleural sparing, (c) emphysema beside the honeycombing area, (d) GGA or reticulation in the honeycombing area, (e) homogeneity of honeycombing, and (f) honeycombing in the upper lobes (Fig 3). Asymmetry was defined as the difference in subsegmental or segmental extent of the FILA between both lower lobes, and if the subsegmental or segmental extent of fibrosis was asymmetric, it was scored 1. Juxta-subpleural sparing was defined as a relative sparing of the immediate subpleural lung in the dorsal regions of the lower lobes [18, 19], and if there was a juxta-subpleural sparing, it was scored 1. The presence of emphysema outside the honeycombing area was observed, and a score of 1 was added if it was present. GGA and reticulation in honeycombing area was defined as the presence of GGA and reticulation just near the honeycombing cysts, absence of GGA or reticulation was scored as 1. Homogeneity was noted when the size and shape of the honeycombing cysts in both lower lobe were uniform, and a score of 1 was given when there was inhomogeneous honeycombing. The presence of honeycombing cysts in upper lobes was also analyzed, and a score of 1 was given if there were no honeycombing cysts in the upper lobes. These 6 scores were totaled to create the “qualitative FILA score (qFILA score)”, which ranged from 0 to 6 in each of the 65 patients.

We also generated another score named “comparative emphysema-fibrosis score (cEMFI score)” to compare the emphysema extent to the fibrosis extent, and this was generated by subtraction of the fibrosis score from the visually assessed emphysema score (Fig 1).

### Clinical Data and Pulmonary Function Tests

Clinical assessments such as clinical characteristics, smoking in pack-years, and pulmonary function tests (PFTs) including forced vital capacity (FVC), forced expiratory volume in 1 second (FEV_1_), FEV_1_/FVC, vital capacity (VC), and the diffusing capacity of the lung for carbon monoxide (DLco) were investigated via review of patient medical records by one of the authors (H.N.J.), who was blinded to the CT evaluation results. PFTs were performed according to the American Thoracic Society guidelines [20], and a portable spirometer (Vmax Spectra 22D, Sensormedics, Yorba Linda, CA, USA) was used. The estimated pulmonary arterial pressure (esPAP) was calculated by adding the transtricuspid pressure gradient with the right atrial pressure [21] by transthoracic Doppler echocardiography. For survival analysis, the date and cause of death were obtained from medical records at our institution and the National Statistical Office of Korea, and the cause of death was classified based on the 10^th^ edition of the International Classification of Diseases (ICD).

### CT Examination

CT scans were performed using a 16 or 128 multi-detector CT scanner (Somatom Sensation 16, Siemens Medical Solution, Erlangen, Germany or Somatom Definition AS Plus, Siemens Medical Solution, Forchheim, Germany, respectively) at end-inspiration in the supine position. Scanning parameters for each scanner were as follows. For the 16-detector row scanner, the detector collimation was 0.75 mm; beam pitch, 1.5; reconstruction thickness, 1.0 mm; reconstruction interval, 10.0 mm; rotation time, 0.75 second; tube voltage, 120 kVp; tube current, 200 effective mAs; and reconstruction kernel, the very sharp algorithm (B70f). For the 64-detector row scanner, the detector collimation was 0.6 mm; beam pitch, 1.0; reconstruction thickness, 1.0 mm; reconstruction interval, 10.0 mm; rotation time, 0.5 second; tube voltage, 120 kVp; tube current, 200 effective mAs; and reconstruction kernel, the very sharp algorithm (B70f). Scanned images were displayed in the lung window setting (window level, –600 to –700 HU; window width, 1200–1500 HU) and were interfaced directly to our Picture Archiving and Communication System (PACS, m-view TM; Marotech, Seoul, Korea).

### Statistical Analysis

Inter-observer agreement for the visually assessed extent of emphysema and FILA, as well as the characteristics of FILA, were analyzed with the weighted kappa test and classified as follows: poor, κ = 0–0.20; fair, κ = 0.21–0.40; moderate, κ = 0.41–0.60; good, κ = 0.61–0.80; and excellent, κ = 0.81–1.00 [22]. Proportions were assessed with 95% confidence intervals (CIs). Comparisons of continuous variables were made with the Student t-test or one-way analysis of variance (ANOVA), and the Chi-square test or Fisher’s exact test was used to compare proportions. Disease-specific survival was defined as the time from baseline CT to the date of death by ILD, with an ICD code of J80-J84. Kaplan-Meier curves were stratified in each group and with two models, which were compared by log-rank test. Conventionally median survival time was extracted, but mean survival time was calculated when the median time could not be estimated because of the small number of occurrences. Univariate Cox proportional hazards regression analysis was performed to identify the prognostic predictors for survival in the UIP without emphysema, UIP with emphysema, and SRIF groups. To discriminate SRIF from UIP with emphysema based on qFILA and cEMFI scores, a logistic regression model with two covariates was constructed, and the optimal cutoff point was individuated in its receiver operating characteristics (ROC) curve according to the Yuden index. “Predictive score for SRIF” was generated with qFILA and cEMFI for a practically simple discrimination of SRIF from UIP with emphysema. Additional details about the process of generating predictive score for SRIF are provided in the S1 File.

Statistical analyses were performed using SPSS version 12.0.1 (SPSS, Chicago, IL) and the R statistical programming environment, version 3.0.2 (the R Foundation, Vienna, Austria) [23]. *P* values < 0.05 were considered significant.

### Ethics Statement

Institutional review board approval was gained for this study from Chonbuk National University Hospital Ethics Committee. The data was analyzed anonymously, and therefore no additional informed consent was required.

## Results

The mean kappa values for the extent of emphysema and FILA for visual assessment had moderate to excellent agreement between the two readers (emphysema: 0.74, reticulation: 0.71, honeycombing: 0.64, total FILA: 0.81). Follow-up periods ranged from 1.25 to 115.63 months, and the median follow-up period was 35.24 months (95% CI: 31.55, 47.25). Thirty-one patients (47.7%) died during follow-up; 17 (17/30, 56.7%) were in the UIP without emphysema group, 13 (13/26, 50.0%) were in the UIP with emphysema group, and 1 (1/9, 11.1%) was in the SRIF group.

### Comparison of Demographics and Survival Analysis between Three Groups

**Fig 4:**
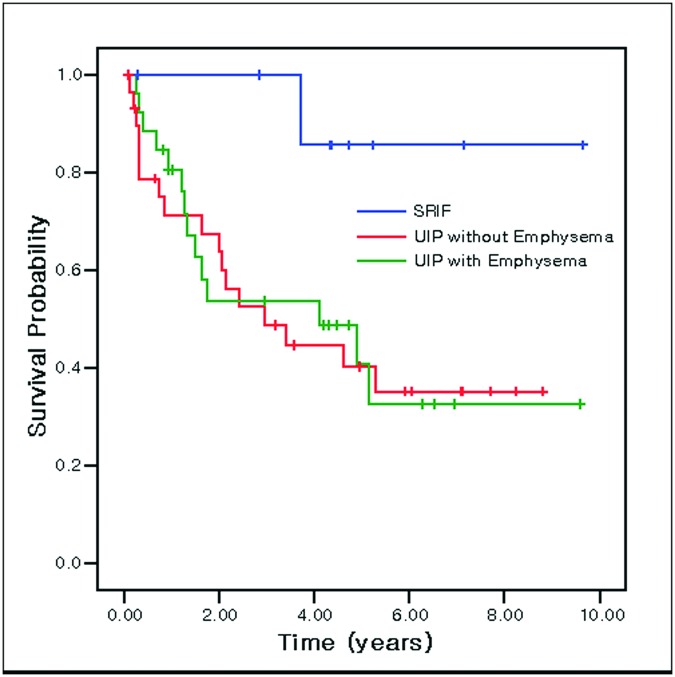
Kaplan-Meier survival curves stratified by three groups, UIP without emphysema, UIP with emphysema and SRIF. The highest survival rate was observed for patients with SRIF (mean survival: 8.79 ± 0.78 years), and this was significantly higher than survival in UIP with emphysema (median survival: 4.10 ± 1.96 years, *p* = 0.035) and UIP without emphysema (median survival: 2.95 ± 1.06 years, *p* = 0.026).

Table 1 shows physiologic characteristics and imaging analysis between three groups (group A: UIP without emphysema, group B: UIP with emphysema, group C: SRIF). Smoking-related changes such as smoking intensity (*p <* 0.001), extent of emphysema (*p <* 0.001) and FVC% (*p* = 0.044) were significantly lower in group A than in the other two groups. However, no factors could differentiate SRIF patients from group B. Kaplan-Meier survival analysis for the three groups is shown in Fig 4. It demonstrated the longest survival for SRIF patients (mean survival: 8.79 ± 0.78 years), while patients in group A (median survival: 2.95 ± 1.06 years) and group B (median survival: 4.10 ± 1.96 years) had a significantly lower survival rate compared to group C (*p* = 0.035 and *p* = 0.026, respectively).

**Table 1:** Comparison of Demographics and Imaging Analysis in UIP without Emphysema, UIP with Emphysema and SRIF Groups with Post-hoc Tests.

	UIP without Emphysema (N = 30)	UIP with Emphysema (N = 26)	SRIF (N = 9)	*P* value	Post-Hoc
**Age**	65.5 ± 7.1	65.8 ± 70.6	67.4 ± 5.8	0.766	
**Sex (M/F)**	16/14	26/0	8/1	<0.001	1,23
**BMI**	23.6 ± 3.7	16.4 ± 1.3	13.3 ± 2.6	0.002	13,2
**Smoking status (never/previous/current)**	17 / 6 / 7	0 / 18 / 8	0 / 6 / 3	<0.002	1,23
**Smoking intensity (PY)**	13.8 ± 20.1	38.3 ± 15.2	37.8 ± 14.0	<0.001	1,23
**FVC%**	79.3 ± 15.4	82.7 ± 13.6	94.2 ± 19.0	0.044	1,23
**FEV**_**1**_**%**	94.7 ± 20.1	92.5 ± 14.9	104.6 ± 19.0	0.216	
**FEV**_**1**_**/FVC**	84.2 ± 4.8	79.7 ± 8.5	79.6 ± 17.3	0.016	1,23
**VC**	80.5 ± 16.3	82.1 ± 15.5	78.4 ± 5.4	0.085	
**RV**	101.9 ± 37.7	112.3 ± 46.4	96.0 ± 22.0	0.728	
**DLco**	70.2 ± 20.0	69.7 ± 20.3	62.0 ± 13.2	0.563	
**esPAP**	36.0 ± 7.4	47.3 ± 15.9	31.0 ± 4.4	0.097	
**Emphysema extent**	0.00	0.96 ± 0.69	1.20 ± 0.42	<0.001	1,23
**Emphysema type (centrilobular/ paraseptal)**	N/A	6: 20	2: 7	1.000	
**Reticulation extent**	1.63 ± 0.81	1.65 ± 0.85	1.22 ±0.44	0.335	
**Honeycombing extent**	1.73 ± 0.87	2.19 ± 1.10	1.78 ±0.83	0.797	
**Total fibrosis extent**	1.93 ± 0.83	2.42 ± 0.99	1.78 ±0.83	0.069	

Data represent the mean ± standard deviation. UIP = usual interstitial pneumonia, SRIF = smoking-related interstitial fibrosis, BMI = body mass index, PY = pack years, FVC = forced vital capacity, FEV_1_ = forced expiratory volume in 1 second, VC = vital capacity, RV = residual volume, DLco = carbon monoxide diffusion capacity, N/A = not available

We evaluated the clinical and radiologic features of each groups and disease-specific survival with Cox proportional hazard regression analysis. No factors affected the survival rate in the SRIF group. However, in group A, a honeycombing extent of 5% or greater was an independent significant indicator for lower disease-specific survival (hazard ratio: 2.871, 95% CI: 1.066–7.731, *p* = 0.037). Similarly, it was a marginal predictor of survival in patients in group B (hazard ratio: 3.362, 95% CI: 0.964–14.679, *p* = 0.057). This data suggests that the survival rate of UIP patients, regardless of whether they have emphysema or not, is lower with 5% or more honeycombing, but that honeycombing does not affect the survival rate of SRIF patients.

### Qualitative Imaging Analysis

Interobserver agreement for 6 qualitative fibrosis scores between two readers ranged from moderate to good (asymmetry: 0.52, juxta-subpleural sparing: 0.61, emphysema besides the honeycombing area: 0.69, GGO/reticulation in honeycombing area: 0.45, homogeneity of the honeycombing area: 0.73, and upper lobe honeycombing: 0.66).

**Fig 5:**
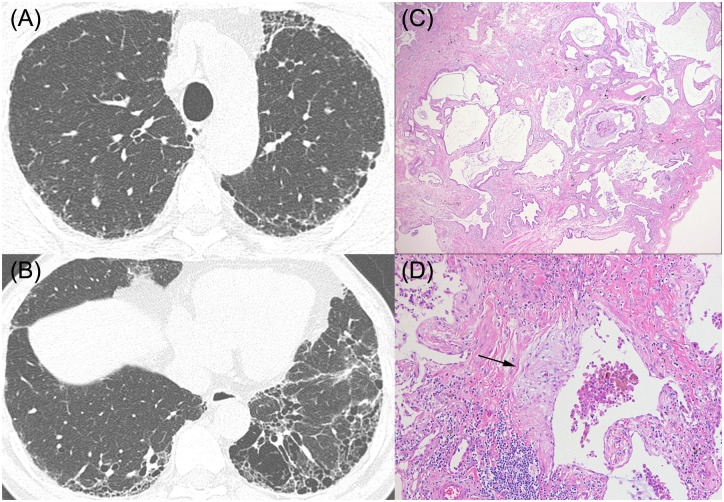
HRCT images and pathologic features of a 66-year-old man who was categorized as UIP without emphysema. (A), (B) HRCT shows subpleural honeycombing predominantly in the lower lobes without emphysema (C), (D) Pathologic features show dense fibrosis with architectural distortion, microscopic honeycomb change (C, x20), and often fibroblastic foci (arrow), (D, x200). He was considered to have idiopathic pulmonary fibrosis, and pSRIF score was -2.

**Fig 6:**
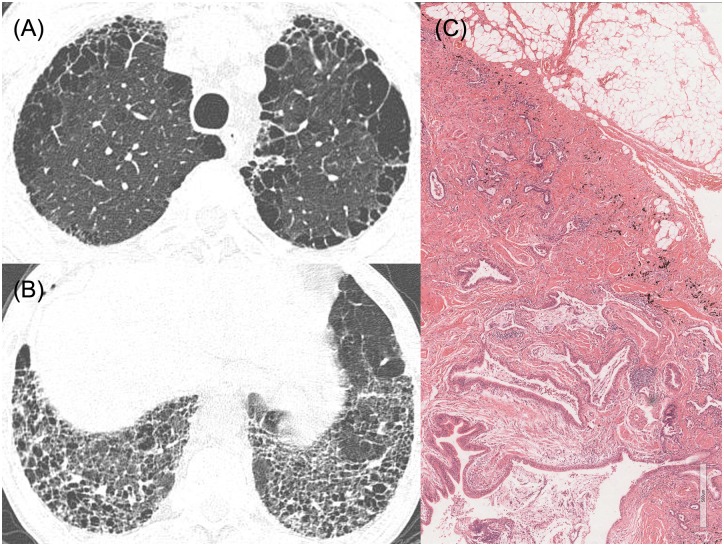
HRCT images and pathologic features of a 64-year-old man who have possible UIP pattern with emphysema. (A), (B) HRCT shows upper lobe-predominant paraseptal emphysema and symmetric honeycombing in lower lobes. (C) Pathologic features show dense fibrosis with severe architectural distortion (x40). He was diagnosed with IPF after multidisciplinary discussion between pulmonologists, radiologists, and pathologists, and the pSRIF score was -3.

**Fig 7:**
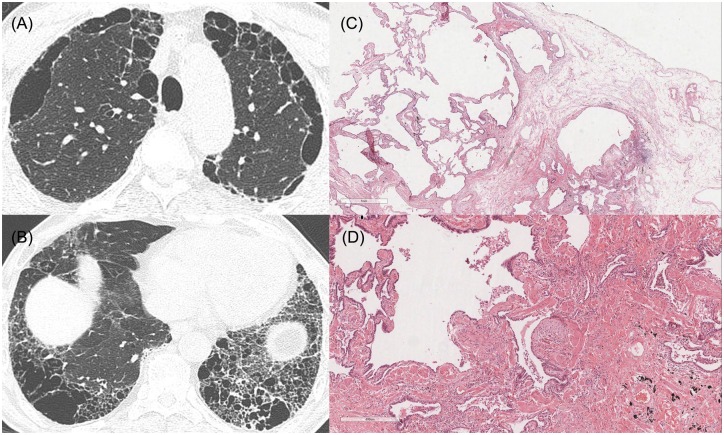
HRCT images and pathologic features of a 67-year-old man who was diagnosed with SRIF in pathology. (A), (B) HRCT shows upper lobe-predominant paraseptal emphysema, and asymmetric, inhomogeneous honeycombing with bullae in the lower lobes. (C, D) Pathologic features show emphysematous change, interstitial fibrosis and dense fibrosis with architectural distortion and fibroblastic focus (x20, x40). He was diagnosed with SRIF by multidisciplinary discussion, because also he was not suspicious to have IPF in clinical information. The pSRIF score was 2.

Details of fibrous interstitial abnormalities between 3 groups and their percentages are shown in Table 2, there was no specific abnormality could distinguish SRIF from UIP with emphysema. The mean values of qFILA and cEMFI for UIP with emphysema group and SRIF group are reported in Table 3. The mean qFILA was 2.19 ± 1.33 in group UIP with emphysema and 3.56 ± 2.07 in group SRIF. The mean cEMFI was -1.46 ± 0.90 in group UIP with emphysema and -0.56 ± 0.88 in group SRIF. The qFILA and cEMFI were significantly higher in group SRIF than in group UIP with emphysema, (*p* = 0.029, 0.014). This means that SRIF patients have more than 3 characteristics among the listed six (asymmetric honeycombing, juxta-subpleural sparing, emphysema besides the honeycombing area, absence of GGA/reticulation in the honeycombing area, inhomogeneous honeycombing, absence of upper lobe honeycombing) and tend to have a higher extent of emphysema compared to patients in the UIP with emphysema group who have a similar FILA extent. “Predictive Score for SRIF (pSRIF score)” defined as qFILA+2×cEMFI (S1 File) was calculated for each patient. The mean values of pSRIF for both groups are also presented in Table 3. The pSRIF for group SRIF is significantly higher than for group UIP with emphysema (*p* = 0.001). This indicates that if a patient has more fibrotic characteristics and has a higher extent of emphysema compared to fibrosis, a SRIF diagnosis is more likely than UIP with emphysema (Figs 5, 6 and 7).

**Table 2:** Comparison of Fibrotic Interstitial Lung Abnormalities on CT in UIP without Emphysema, UIP with Emphysema and SRIF Groups.

	UIP without Emphysema (N = 30)	UIP with Emphysema (N = 26)	SRIF (N = 9)
**Asymmetry**	10 (33.3)	10 (38.5)	6 (66.7)
**Juxta-subpleural sparing on honeycombing area**	3 (10)	6 (23.1)	4 (44.4)
**Emphysema beside the honeycombing area**	1 (3.3)	11 (42.3)	5 (55.6)
**Absence of ground grass attenuation/reticulation in honeycombing area**	10 (33.3)	5 (19.2)	4 (44.4)
**Inhomogeneous honeycombing**	3 (3.3)	9 (34.6)	6 (66.7)
**Absence of honeycombing in the upper lobes**	15 (50)	16 (61.5)	7 (77.8)

Parentheses represent percentage. UIP = usual interstitial pneumonia, SRIF = smoking-related interstitial fibrosis.

**Table 3:** Comparison of CT Scores between UIP with Emphysema and SRIF Groups.

	UIP with Emphysema (n = 26)	SRIF (*n* = 9)	*P* Value
**qFILA score**	2.19 ± 1.33	3.56 ± 2.07	.029
**cEMFI score**	-1.46 ± 0.90	-0.56 ± 0.88	.014
**Predictive score for SRIF**	-0.73 ± 1.95	2.44 ± 2.96	.001

Data are represent the mean ± standard deviation. UIP = usual interstitial pneumonia, CPFE = combined pulmonary fibrosis and emphysema, qFILA score = score generated by adding the 6 CPFE patterns from Fig 3, cEMFI score = subtraction of fibrosis score from emphysema score visually assessed refer to Fig 1, “predictive score for CPFE” = (qFILA)+(2×cEMFI).

## Discussion

This is the first trial study that attempts to differentiate SRIF from UIP with emphysema by quantitative and qualitative imaging analysis with pathologically proven cases. In our study, pathologically proven UIP patients with or without emphysema had higher mortality than those with SRIF. This result seems to contradict other studies; Mejia et al. [8] reported that patients with idiopathic pulmonary fibrosis (IPF) and emphysema had a higher mortality rate than patients with IPF without emphysema. Sugino et al. [10] also pointed out that survival was significantly worse in patients with CPFE than in those with IPF alone, despite exclusion of lung cancer-related deaths. On the other hand, mortality was similar in patients with CPFE and IPF in a study that defined CPFE as ≥10% emphysema. We believe that these inconsistent results may be affected by the imprecise differentiation of CPFE from IPF, as well as the heterogeneous study populations involved [17]. We only included patients with pathologically proven UIP and SRIF which exhibited emphysema with alveolar septal widening by collagen deposition without evidence of UIP. Biopsy proven UIP patients were diagnosed as IPF because they were clinically, radiologically and pathologically met the criteria of IPF. However, for the cases of SIRF, they were not considered with IPF after the multidisciplinary discussion between pulmonologists, radiologists and pathologists. And as their survival rates were quite different, the effort to differentiate SRIF from UIP with emphysema among CPFE patients would be necessary. Through our study, we firmly believe that the concept of differentiating SRIF from UIP with emphysema by radiologic findings not by the invasive procedure is attempted.

SRIF was firstly termed by Katzenstein et al. [5], which is characterized by uniform thickening of alveolar septa by collagen deposition with minimal associated inflammation, and accompanying emphysema. This is distinguished from UIP by hyalinized, eosinophilic collagen deposition that variously thickens alveolar septa, and enlarged airspaces of emphysema as well as RB. In contrast, UIP is characterized by the combination of a patchwork pattern of lung involvement by the fibrosis, and fibroblast foci are more frequently seen [24]. This fibroblastic foci represent an area of acute lung injury, thus a widely accepted pathogenesis of UIP is a focal acute lung injury while the etiology of lung injury is still uncertain [25]. In SRIF, the fibroblastic foci can be found but they are small and inconspicuous, moreover it usually is combined with emphysema and RB. This represents SRIF is developed by smoking, and the fibrosis would be less active and progressive. Our data support this hypothesis and we emphasize the necessity of differentiating SRIF and UIP with emphysema among the CPFE patients.

In our study, there were no demographic or physiologic factors differentiating SRIF from UIP with emphysema, and only the FILA characterizations were helpful. Emphysema besides the honeycombing area, juxta-subpleural sparing of honeycombing, asymmetric and/or inhomogeneous honeycombing, absence of GGA-reticulation in honeycombing, and absence of honeycombing in the upper lobes are more likely to be seen in SRIF patients. Recently, CPFE was recognized as a coexisting pattern in the American Thoracic Society/European Respiratory Society criteria for the diagnosis of IIP [1]; thus, this could include not only the CT pattern of UIP or fibrosing NSIP, but also SRIF. In addition, recent studies described the radiologic and pathologic findings of thick-walled cystic lesions as a kind of features in CPFE [7, 26]. This corresponds to some of our FILA characterizations, such as emphysema besides the honeycombing area or asymmetric, inhomogeneous honeycombing cysts. The pathogenesis of SRIF is unknown, but we suggest that inflammatory or other unknown processes derived from smoking could impact emphysema and fibrosis. Thus, these interactive and/or additive effects could develop asymmetric and inhomogeneous honeycombing, and also there can be emphysema besides the honeycombing areas. Differentiating these patterns is critical to reduce unnecessary invasive procedures, and could predict patient prognosis as well.

There were several efforts to differentiate CPFE from IPF, many of them restricted emphysema extent for distinction. Ryerson et al. [11] defined CPFE as ≥ 10% of emphysema, and this has clinical relevance for identifying Global Initiative for Chronic Obstructive Lung Disease (GOLD) stage II equivalent disease. Kitaguchi et al. [27] suggested a threshold of ≥ 25% of emphysema extent for increased specificity. These efforts came from the hypothesis that smoking is a strong causative factor for CPFE whether IPF is idiopathic, consensus definition of CPFE does not currently exist. We included patients as CPFE who have visible emphysema on CT scan according to Cottin et al [3], and among the CPFE patients we tried to differentiate smoking-related fibrosis from UIP by comparative emphysema extent named cEMFI score. This was generated by subtraction of fibrosis extent from emphysema extent. Patients with SRIF were likely to have higher cEMFI scores, whether there was no difference when we simply compared emphysema extent between SRIF and UIP with emphysema. This suggests that emphysema extent is higher in SRIF patients compared to those with emphysematous UIP who have the same extent of fibrosis. Thus not only the absolute extent of emphysema but also the relative extent of emphysema to fibrosis would be helpful for distinguishing SRIF from UIP with emphysema.

This study has several limitations. First, this was a retrospectively designed study at a single center, and the number of included patients was relatively small. Additional prospectively designed studies with a larger sample size are warranted to support our results. Second, both emphysema and fibrosis extent were visually assessed. Quantification could give us objective data, but in the current quantification method, honeycombing cysts could be involved in a low attenuation area (LAA%), which indicates emphysema extent. Because the quantified emphysema extent could be larger in both SRIF and UIP with emphysema patients and the comparison is therefore limited, we used visual analysis by two radiologists, whose agreement was moderate to excellent. Third, although we only included pathologically proven data, there was only a single biopsy site, which could lead to selection bias. However, we specifically selected a biopsy site that could possibly combine emphysema, honeycombing cysts and some GGA. Fourth, 55.6% (5/9) of SRIF patients, 92.3% (24/26) of UIP with emphysema patients and 86.7% (26/30) of UIP without emphysema patients were treated with corticosteroids, which could potentially have influenced the survival rate. However, this was likely only a small effect because we analyzed disease-specific survival rate and excluded deaths related to infection.

## Conclusion

In conclusion, it is not easy to distinguish SRIF from UIP with emphysema among CPFE patients, but we should try to differentiate two diseases because their survival rate could be different. Especially, having more than three fibrotic CT patterns (emphysema besides honeycombing area, juxta-subpleural sparing of honeycombing, inhomogeneous honeycombing, absence of honeycombing in the upper lobes, asymmetric honeycombing, and absence of GGA/reticulation in the honeycombing area) could predict favorable outcomes in CPFE patients. Although further studies with a larger patient population are needed, this CT assessment of fibrotic patterns may contribute to the differentiation of SRIF from similar conditions, and eventually might reduce unnecessary therapy or procedures.

## Supporting Information

S1 Fig ROC curve for logistic regression. Graph shows the ROC curve for the logistic regression model and the optimal cutoff value 0.3 is highlighted with the relative values of sensitivity (77.8%) and specificity (84.6%).
(TIF)

S2 Fig Probability calculation. The (qFILA, cEMFI) points are indicated at which the estimated CPFE probabilities are higher than the optimal cutoff value of 0.3 and the solid black line depicts the equation qFILA+2×cEMFI = 2.
(TIF)

S1 File Predictive Score for SRIF (pSRIF score). (DOCX)

S1 Table Logistic Regression Coefficients. (DOCX)
