# Supplementary figures and images for: Differentiating Smoking-Related Interstitial Fibrosis (SRIF) from Usual Interstitial Pneumonia (UIP) with Emphysema Using CT Features Based on Pathologically Proven Cases
Source: PLoS One. 2016 Sep 9;11(9):e0162231. doi: 10.1371/journal.pone.0162231 (PMC5017577; doi:10.1371/journal.pone.0162231)

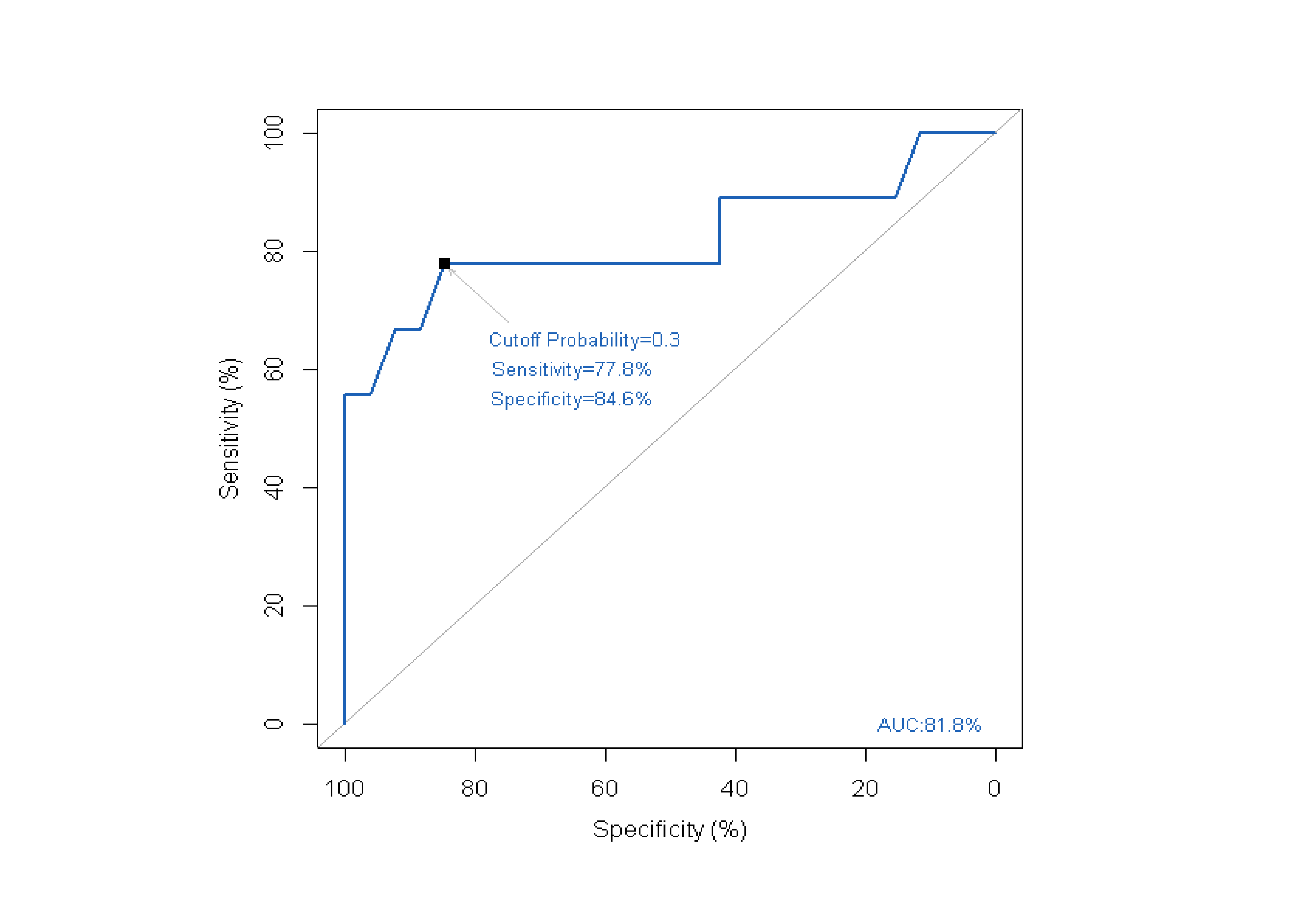

Supplement: S1 Fig — Graph shows the ROC curve for the logistic regression model and the optimal cutoff value 0.3 is highlighted with the relative values of sensitivity (77.8%) and specificity (84.6%). (TIF) [file pone.0162231.s001.tif]

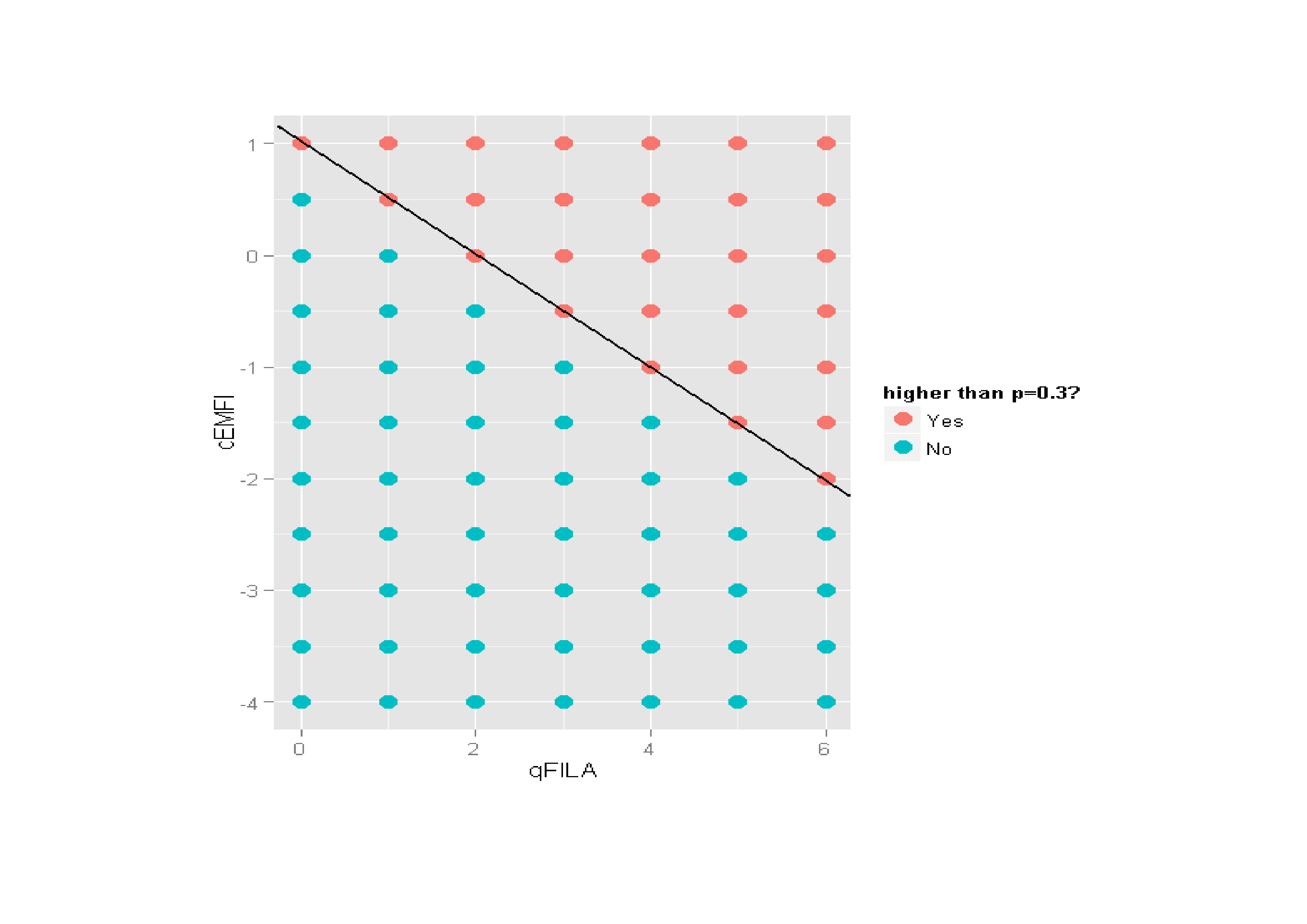

Supplement: S2 Fig — The (qFILA, cEMFI) points are indicated at which the estimated CPFE probabilities are higher than the optimal cutoff value of 0.3 and the solid black line depicts the equation qFILA+2×cEMFI = 2. (TIF) [file pone.0162231.s002.tif]
