# Supplementary material for: Differentiating Smoking-Related Interstitial Fibrosis (SRIF) from Usual Interstitial Pneumonia (UIP) with Emphysema Using CT Features Based on Pathologically Proven Cases
Source: PLoS One. 2016 Sep 9;11(9):e0162231. doi: 10.1371/journal.pone.0162231 (PMC5017577; doi:10.1371/journal.pone.0162231)
Supplement: S1 File — (DOCX) [file pone.0162231.s003.docx]

“Predictive Score for SRIF (pSRIF score)” defined as qFILA+2×cEMFI was proposed for a discriminator between SRIF and UIP with emphysema in the following.

To differentiate SRIF from UIP with emphysema by using qFILA and cEMFI scores, a logistic regression model was constructed for the SRIF probability estimation with two covariates as follows (**S1 Table**):

$$\log\frac{P(SRIF)}{1-P(SRIF)}=-1.460+\left( 0.548\times qFILA score \right)-(1.171\times cEFMIscore)$$

Equivalently, the probability of SRIF for each (qFILA, cEFMI) pair was estimated as

$$\frac{1}{1+exp\{1.460-\left( 0.548\times qFILA score \right)+\left( 1.171\times cEFMIscore \right)\}}$$

The receiver operating characteristics (ROC) curve for the model is shown in **S1 Fig.**  The area under the ROC curve (AUC) was 0.818. The optimal cutoff value of 0.3 was individuated in the ROC according to the Yuden index. At an optimal probability cutoff of 0.3, there was a sensitivity of 77.8% and specificity of 84.6 for identification of SRIF. For a differentiation between SRIF and UIP with emphysema based on the constructed regression model, the calculation of the SRIF probability for each patient is required but is not practically trivial in the clinical applications. To resolve this limitation, the probability calculation was implemented for every possible pair of (qFILA, cEMFI) and the score pairs with probabilities of 0.3 or greater are indicated in **S2 Fig**. The SRIF probability is higher than the optimal cutoff for (qFILA, cEMFI) satisfying qFILA+2×cEMFI≥2 and lower elsewhere. From this observation, “Predictive Score of SRIF (pSRIF)” defined as qFILA+2×cEMFI was developed. It would be generated by relatively simple computation and used for a discriminator between SRIF and UIP with emphysema that patients with pSRIF scores≥2 are diagnosed with SRIF.
