# Supplementary material for: Differentiating Smoking-Related Interstitial Fibrosis (SRIF) from Usual Interstitial Pneumonia (UIP) with Emphysema Using CT Features Based on Pathologically Proven Cases
Source: PLoS One. 2016 Sep 9;11(9):e0162231. doi: 10.1371/journal.pone.0162231 (PMC5017577; doi:10.1371/journal.pone.0162231)
Supplement: S1 Table — (DOCX) [file pone.0162231.s004.docx]

**S1 Table. Logistic Regression Coefficients**

|  | **Coefficient Estimate** | **Standard Error** |
| --- | --- | --- |
| **Intercept** | -1.460 | 1.102 |
| **qFILA score** | 0.548 | 0.293 |
| **cEMFI score** | 1.171 | 0.568 |

_
